# Identification of new DNA-associated proteins from *Waddlia chondrophila*

**DOI:** 10.1038/s41598-019-40732-1

**Published:** 2019-03-20

**Authors:** Marie de Barsy, Lucas Herrgott, Virginie Martin, Trestan Pillonel, Patrick H. Viollier, Gilbert Greub

**Affiliations:** 1 Institute of Microbiology, University Hospital Center and University of Lausanne, Lausanne, Switzerland; 2 Department Microbiology and Molecular Medicine, Institute of Genetics & Genomics in Geneva (iGE3), University of Geneva, Geneva, Switzerland

**Keywords:** Bacteria, Transcriptional regulatory elements

## Abstract

Transcriptional regulation in *Chlamydiae* is still poorly understood. The absence until recently of genetic tools is the main cause of this gap. We discovered three new potential DNA-associated proteins of *Waddlia chondrophila*, a *Chlamydia*-related bacterium, using heparin chromatography coupled to mass spectrometry (Wcw_0377, Wcw_1456, and Wcw_1460). By ChIP-seq analysis, we determined the regulatory landscape of these three proteins and we showed that Wcw_0377 binds all along the genome whereas Wcw_1456 and _1460 possess a wide regulon with a large number of co-regulated genes. Wcw_1456 and Wcw_1460 interact with RpoD (σ^66^), emerging as potential RpoD regulators. On the other hand, Wcw_0377 is able to reach the host nucleus, where it might interact with eukaryotic histones through its putative chromatin-remodelling SWIB/MDM2 domain.

## Introduction

Transcription regulation is a key process for all organisms. In bacteria, transcriptional regulation occurs at three different levels: (i) DNA structure (nucleoid-associated proteins, histone-like proteins), (ii) RNA polymerase (sigma factors) and (iii) promoter (transcription factors)^1^. Transcriptional regulation is a complex process well characterized in model organisms such as *E. coli*^2,3^. Due to their reduced genome (that encodes fewer sigma factors and transcription factors), chlamydiae bacteria are considered as ideal organisms to elucidate the transcriptional regulatory network. All bacteria members of the *Chlamydiae* phylum share a biphasic, temporally regulated developmental cycle characterized by the “infectious and non-dividing” elementary bodies (EBs) and the “dividing” reticulate bodies (RBs)^4–6^. The EB enters into a host cell, resides in a vacuole called inclusion and differentiates into RB. The RBs multiply by several binary fissions and differentiate back into EBs that are released after cell lysis. This cycle is regulated by three temporal waves of gene expression, known as early-phase genes (expressed during the entry and the differentiation), mid-phase genes (expressed during the multiplication) and late-phase genes (expressed during the re-differentiation and the release). In addition, EBs and RBs are easily typified by the nucleoid condensation observed in the EBs and the dispersed chromatin in RBs. This nucleoid condensation is unique among prokaryotes. Most studies focussed on the best-known pathogens, *Chlamydia trachomatis*, the world’s most common sexually transmitted bacterial pathogen, and *Chlamydia pneumoniae*, responsible of lung infections^7–9^. It was reported that *C. trachomatis* possesses two histone H1 homologs, called Hc1 and Hc2 (encoded by *hctA* and *hctB* genes respectively), that are involved in the DNA condensation in the EBs^10–13^. It is thought that nucleoid condensation in EBs is responsible for a global transcriptional down-regulation likely due to the inaccessibility of the genomic DNA to the RNA polymerase. Nevertheless, the exact mechanism of nucleoid condensation and its regulation are still poorly understood. Recently, it was reported that DNA supercoiling increases at mid-phase and that many mid-phase genes are responsive and transcriptionally activated by this change in DNA supercoiling^14–16^.

*Chlamydia trachomatis* encodes three sigma factors, σ^66^ (CT615), σ^54^ (CT609) and σ^28^ (CT061) that could direct RNA polymerase (RNAP, E) to specific promoter sequences. Based on homology to *E. coli* σ^70^, σ^66^ of *C. trachomatis* is considered as the major sigma factor, expressed all along the developmental cycle^17^, while σ^28^ is temporally regulated^17^ and activates several late-phase genes^18,19^. Little is known concerning σ^54^. Only two promoters were reported to be bound by σ^54 ^^20^. The promoter selectivity is conferred by sigma factor-bound RNAP (Eσ). Transcriptional activation may be further fine-tuned by transcriptional activators or repressors that target motifs in or near the core promoters (i.e. the determinants recognized by Eσ). At least ten transcription factors (TFs) are encoded in all known members of the *Chlamydiae* phylum^21,22^, including the master regulator Euo^23–25^ and the heat shock-repressor HrcA^26,27^. Recently, the regulatory landscape of ten conserved TFs of *Waddlia chondrophila* was determined and Euo was shown to bind at least 100 distinct loci, mostly in putative promoter sequences^21^. However, the number of chlamydial TFs that were identified based on sequence homology with known TFs from other bacteria is low compared to the around 300 TFs identified in free-living bacteria such *E. coli*^28^. Moreover, the genetic basis of transcriptional regulation during the chlamydial developmental cycle is still poorly understood as well as the DNA structure and topology that have a direct impact on transcription. To fill this gap and to improve our understanding of the regulation of the developmental cycle by these strict intracellular bacteria, we identified new DNA-associated proteins that might be involved in transcriptional regulation using heparin chromatography^29,30^. We applied this innovative approach to a *Chlamydia*-related bacterium, *Waddlia chondrophila*, an emerging pathogen responsible for abortion in ruminants and adverse pregnancy outcomes in humans^31–34^.

## Results

### Identification of three potential DNA-associated proteins

**Figure 1:**
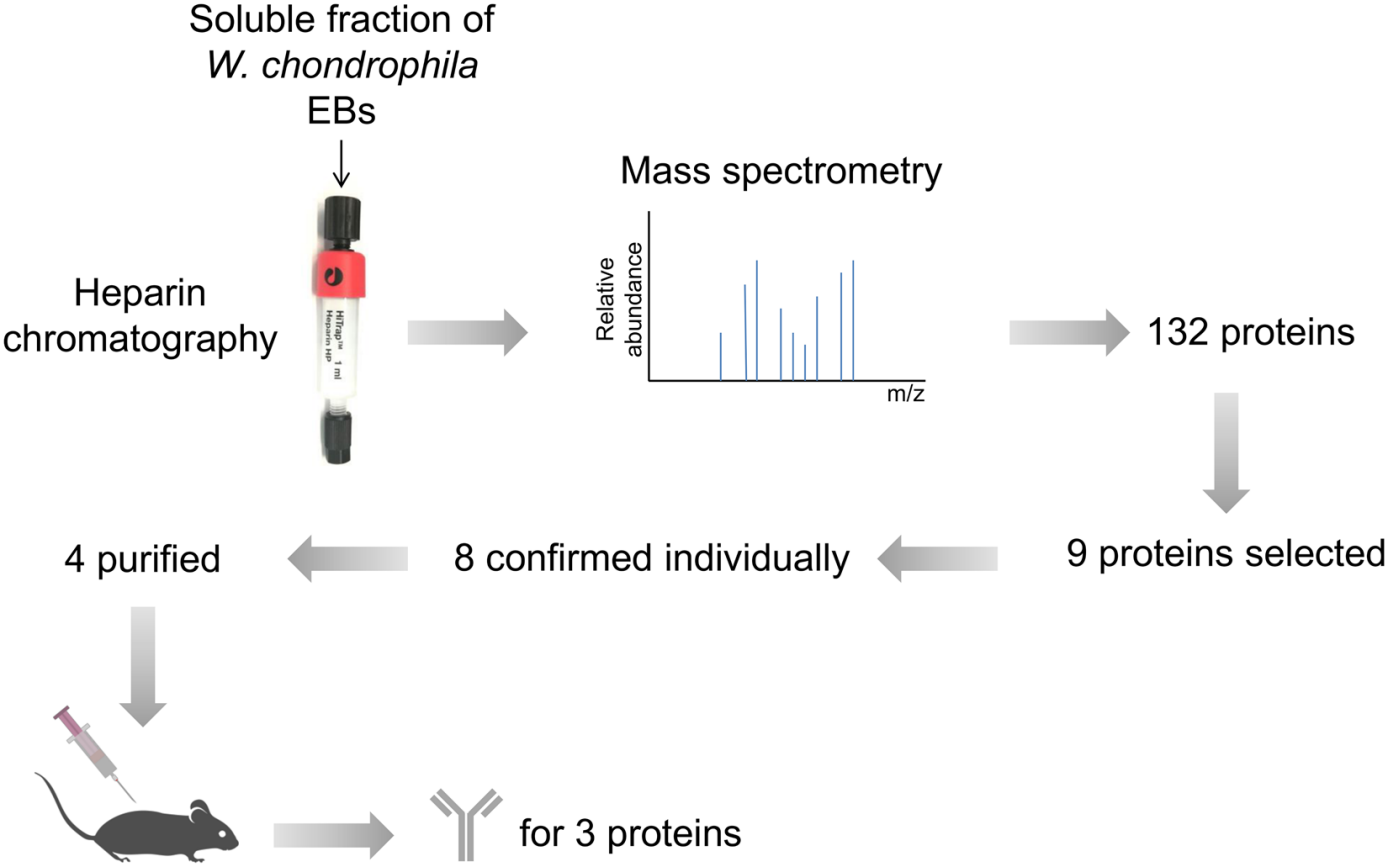
Complete procedure to identify new *Waddlia* DNA binding proteins.

In order to identify potential DNA-associated proteins of *W. chondrophila*, we performed a heparin affinity-chromatography, which is known to trap DNA-associated proteins due to its structure and global negative charge mimicking DNA. The soluble fraction of purified elementary bodies (EBs) was applied to heparin chromatography followed by elution with increasing salt (NaCl) concentrations and mass spectrometry analysis to identify heparin retained proteins. We identified 132 enriched proteins (with a fold change ≤ 0.5 with 95% confidence; Fig. 1 and Supplementary Table S1). Since we wanted to identify new DNA-associated proteins, we first selected only those with unknown function in bacteria. Second, we focused our attention on hypothetical proteins combining the three following criteria, (i) a basic isoelectric point, (ii) harbouring a domain potentially involved in DNA binding and (iii) conserved at least in *Chlamydiales* order. This selection led to nine candidate proteins (Supplementary Table S2). Eight of them could be produced as His-tagged variants in *E. coli* and their individual ability to bind the heparin-matrix could be confirmed (Supplementary Fig. S1). Four His-tagged proteins were purified and effective mouse polyclonal antibodies to three of them (Wcw_0377, Wcw_1456, Wcw_1460 from the NCBI entry CP001928) were obtained. Wcw_0377 harbours a SWIB/MDM2 domain that belongs to the SWI/SNF family of complexes, which are ATP-dependent chromatin-remodelling proteins involved in transcriptional activation^35^. Unfortunately, SWIB/MDM2 domain is not well characterized and the residues involved in DNA-binding are not known. The two other proteins possess a conserved domain of unknown function. Nevertheless, the chlamydial homolog of Wcw_1456 (CT398) was recently shown to interact with RpoN as well as with two Type III secretion ATPase regulators, CdsL and FliH^36^, while the chlamydial homolog of Wcw_1460 (CT429) was described as an effector secreted by the Type Three Secretion System in the *Yersinia enterocolitica* heterologous system^37^.

### Wcw_0377, Wcw_1456 and Wcw_1460 are expressed during the mid-phase

**Figure 2:**
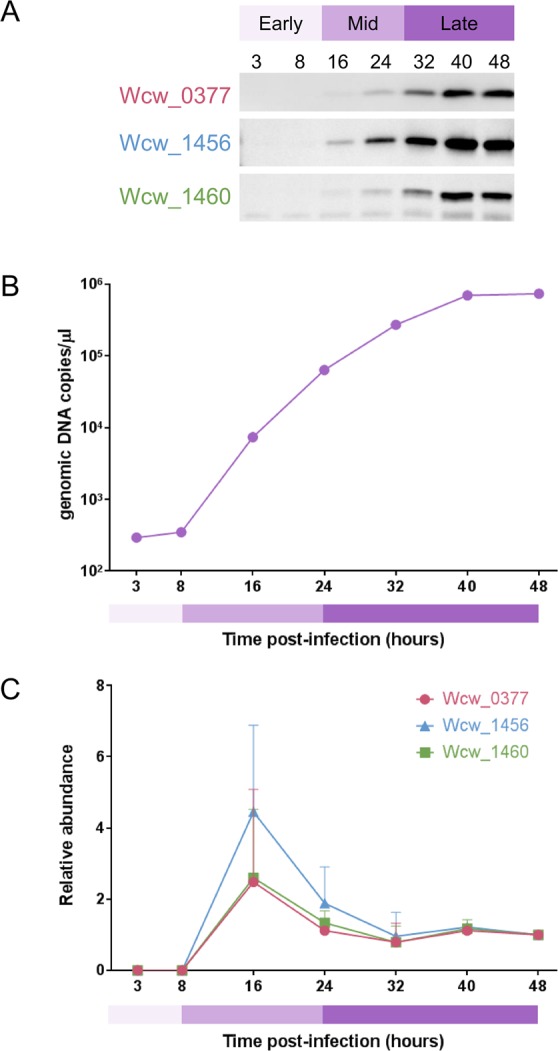
Temporal expression of the three *Waddlia* proteins during the developmental cycle. (**A**) Samples of infected Vero cells were collected at different times p.i. and analysed by immunoblotting using specific mouse polyclonal antibodies (blots were cropped and grouped, see Supplementary Fig. S2 for complete blot pictures). (**B**) In parallel, genomic DNA was extracted and the *Waddlia* specific qPCR was performed to determine the genomic DNA copy per µl and infer the number of bacteria per well. (**C**) Relative abundance of each protein was quantified by normalization of the signal detected on the blot according to the number of bacteria/well defined by qPCR. All the three protein peaked at 16 h p.i., then decrease and remain stable between 32 and 48 h p.i. Panels (A) and (B) are representative results, Panel (C) is an average of three independent experiments.

To determine the relative abundance of these three proteins during the different phase of the *W. chondrophila* developmental cycle, we conducted immunoblotting using mouse polyclonal antibodies on samples of infected Vero cells collected at various times following. The relative protein abundance peaked at 16 h post infection (p.i.) for all three proteins, and then decreased and remained relatively stable until the end of the developmental cycle (Fig. 2 and Supplementary Fig. S2). These result suggests that these three proteins may be involved in regulating the expression of mid-phase genes such those involved in the multiplication of *W. chondrophila*.

### Wcw_0377, Wcw_1456 and Wcw_1460 co-localize with bacterial DNA in aberrant bodies

**Figure 3:**
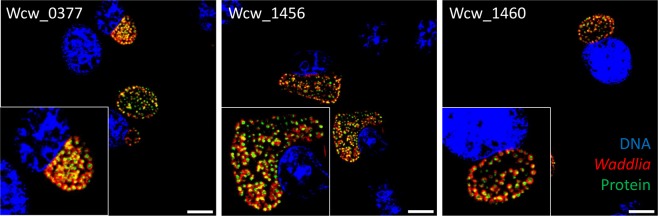
Localization of Wcw_0377, _1456 and _1460 in infected Vero cells. Infected Vero cells were fixed at 24 h p.i. and proteins were detected by immunofluorescence using specific mouse polyclonal antibodies to the three proteins (green). *W. chondrophila* was detected using a rabbit polyclonal antibody anti-*Waddlia* (red) and DNA stained with DAPI (blue). All the three proteins showed a bacterial cytoplasmic localization. Scale bar: 10 µm.

**Figure 4:**
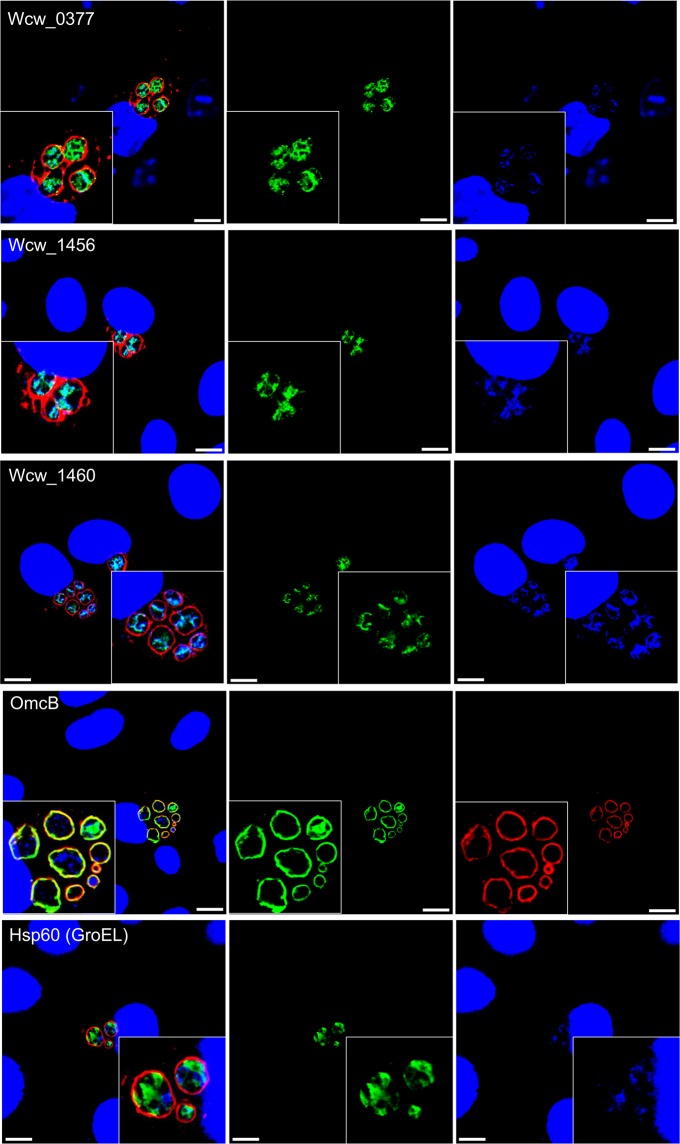
Co-localization of Wcw_0377, _1456 and _1460 with bacterial DNA in *W. chondrophila* aberrant bodies. Infected Vero cells were treated with phosphomycin (500 µg/ml) from 2 h until 24 h p.i. to induce the formation of *W. chondrophila* aberrant bodies. Infected cells were fixed, proteins detected using specific mouse polyclonal antibodies to the three proteins (green), *W. chondrophila* using a rabbit polyclonal antibody anti-*Waddlia* (red) and DNA stained with DAPI (blue). All the three proteins co-localized with bacterial DNA. Scale bar: 10 µm.

We next used immunofluorescence to determine the localization of Wcw_0377, Wcw_1456 and Wcw_1460. All three proteins were localized in the bacterial cytoplasm 24 h after infection of Vero cells with *W. chondrophila* (Fig. 3). As it was not possible to assess whether the three proteins co-localized with DNA due to the small size of the bacteria, we induced the formation of aberrant bodies (ABs), which are enlarged bacteria produced under diverse stresses such as interferon gamma or antibiotics^38,39^. To induce the formation of ABs, infected Vero cells were treated with phosphomycin from 2 h p.i. to 24 h p.i. (22 h of treatment). The DNA stained by DAPI was more visible in ABs. All three proteins co-localized with the bacterial DNA (Fig. 4). OmcB and Hsp60 that localize respectively in the outer membrane and in the bacterial cytoplasm were used as negative controls. These results strongly suggest that Wcw_0377, Wcw_1456 and Wcw_1460 directly or indirectly bind bacterial DNA.

### ChIP-seq reveals large regulatory landscapes for Wcw_1456 and Wcw_1460

**Figure 5:**
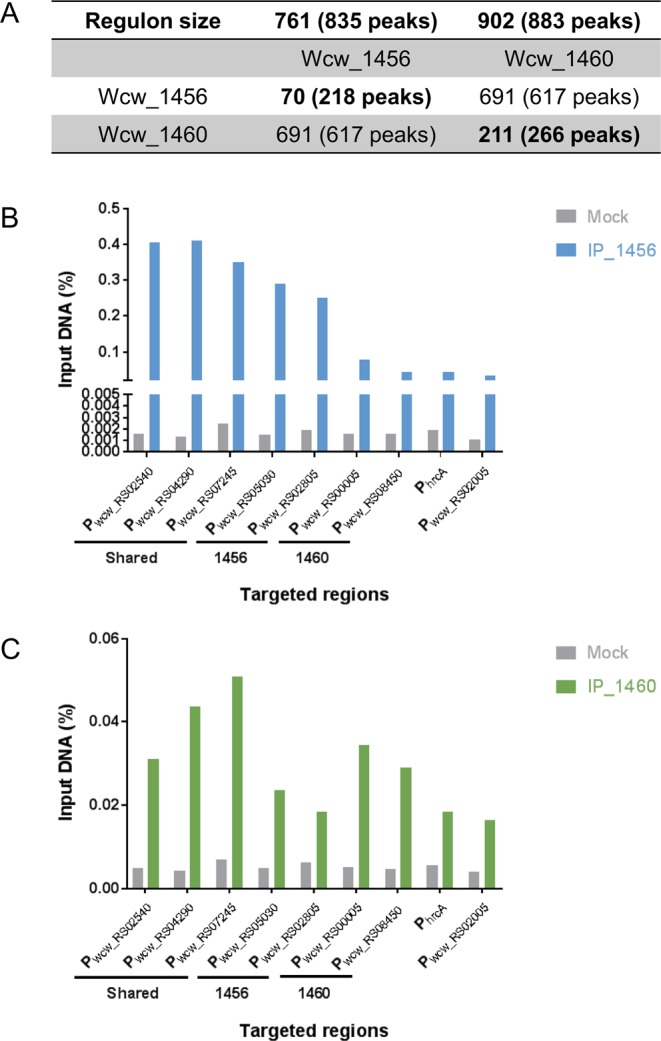
Wcw_1456 and _1460 regulons and targets validation by ChIP-qPCR. (**A**) Each peak identified by ChIP-Seq was assigned to its potentially regulated gene(s). ChIP-Seq analysis showed that Wcw_1456 regulates 761 genes while Wcw_1460 regulates 902 genes, with 691 co-regulated genes. (**B**,**C**) Validation of the regulated genes by ChIP-qPCR. Experiments were performed independently two times and representative results were shown on panel (B) and (C). Primers pairs targeting promoter regions were designed for three co-regulated genes, two Wcw_1456 specific genes and two Wcw_1460 specific genes. As control, primers for the *hrcA* and *wcw_RS02005* promoters were used. (**B**) As expected, binding of Wcw_1456 was confirmed for the three shared regions and the two regions specifically bound by Wcw_1456. (**C**) The binding was also confirmed for Wcw_1460 with the shared regions and the Wcw_1460 specific regions.

To confirm DNA binding and to elucidate the regulatory landscape of the Wcw_1456 and Wcw_1460 proteins, we infected Vero cells with *W. chondrophila* and recovered the infected cells at 48 h post infection (p.i.) to perform ChIP-Seq using specific mouse polyclonal antibodies. We identified 835 peaks for Wcw_1456 and 883 peaks for Wcw_1460 (Fig. 5A and Supplementary Table S3; >100 bp, p value ≤ 0.01).

In order to identify putative promoter regions regulated by Wcw_1456 and Wcw_1460, We assigned ChIPseq peaks to the neighbouring genes (max of the peak located in a window of 400 nt before and 100 nt after the gene start). We thus defined a regulon of 761 genes for Wcw_1456 and 902 genes for Wcw_1460, with 691 co-regulated genes. Seventy genes were specifically regulated by Wcw_1456 and 211 by Wcw_1460 (Fig. 5A and Supplementary Table S4). Three shared targets and two specific targets for each protein were chosen among those with the highest peak shape score and specific primer pairs were designed to perform ChIP-qPCR (see Methods). As negative control, we used the P_hrcA_ and P_wcw_RS02005_ promoters. Vero cells were infected with *W. chondrophila* and harvested at 48 h p.i. to perform chromatin immunoprecipitation using mouse polyclonal anti-Wcw_1456/anti-Wcw_1460 antibodies or no antibody (mock control). For immunoprecipitation using anti-Wcw_1456, we observed an increased binding for the shared target regions as well as the Wcw_1456 specific target regions compared to the Wcw_1460 specific regions and the negative controls (Fig. 5B). We observed a similar result for Wcw_1460, with an increased binding for the shared target regions and the Wcw_1460 specific target regions (Fig. 5C). Taken together, these results confirmed the binding of these two proteins to three shared and two specific targets for each protein.

**Figure 6:**
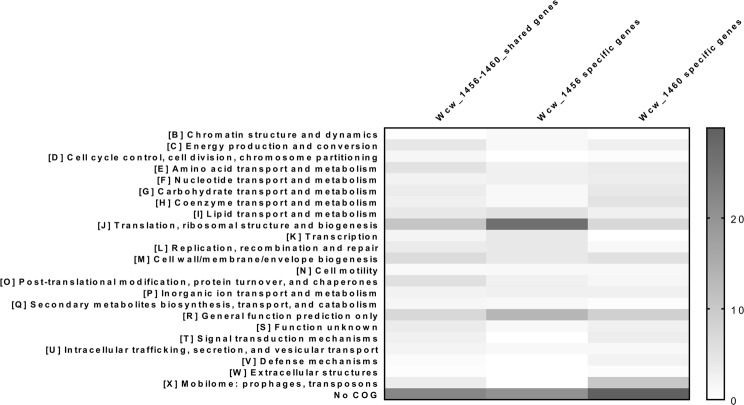
COG categories regulated by Wcw_1456 or Wcw_1460 and co-regulated. The percentage of each COG category among genes specifically regulated by Wcw_1456 or Wcw_1460, as well as for co-regulated genes, was determined. Wcw_1456 mainly regulates genes involved in translation, ribosomal structure and biogenesis, while Wcw_1460 mainly regulates genes involved in the mobilome.

Finally, we proceeded to a COGs analysis to shed light on possible function(s) of these two proteins. Wcw_1456 mainly binds promoter of genes involved in translation, as well as ribosomal structure and biogenesis (COG J, Fig. 6), while Wcw_1460 seems to be involved in the regulation of the mobilome (prophages and transposons, COG X, Fig. 6).

### RpoD as a potential interacting protein of Wcw_1456 and _1460

The regulon size of Wcw_1456 and Wcw_1460 is in the range of regulons of sigma factors such as RpoD (σ^66^) and RpoN (σ^54^)^40^, raising the possibility that these proteins could be associated to sigma factors. The large number of co-regulated genes indicates that Wcw_1456 and Wcw_1460 could also interact with each other. We thus performed a His pull-down followed by mass spectrometry analysis to identify potential interactors of these two proteins (His_6__Wcw_1456 and His_6__Wcw_1460). His-tagged recombinant proteins produced in *E. coli* were immobilized on cobalt resin and the soluble proteins of purified EBs were applied on the resin. The protein complexes were eluted by addition of SDS sample buffer and analysed by mass spectometry. *E. coli* expressing the His tag alone was used as control. Twenty four *Waddlia* proteins were identified in this pull-down assay (Supplementary Table S5), RpoD being the only one interacting with the two recombinant proteins (His_6__Wcw_1456 and His_6__Wcw_1460) and not detected in the negative control. We thus considered RpoD as a potential interactor of Wcw_1456 and Wcw_1460. We did not detect any interaction between Wcw_1456 and Wcw_1460.

### SWIB/MDM2 domain present in Wcw_0377 is highly conserved in *Chlamydiae* phylum

ChIP-Seq performed with anti-Wcw_0377 antibodies identified 1920 peaks (as many as the genes annotated on *Waddlia* genome), suggesting that Wcw_0377 binds DNA all along the *Waddlia* genome, which is consistent with the presence of a SWIB/MDM2 domain potentially involved in chromatin-remodelling. Due to the ability of this protein to spread all along the genome, we did not analyze further the ChIPseq results and focused on the SWIB/MDM2 domain.

We analysed the taxonomic distribution of the SWIB/MDM2 domain present in Wcw_0377 and showed that it is highly conserved among the *Chlamydiae* phylum (100% of bacteria in this phylum) and also present in *Verrucomicrobia* (60%) as well as in some *Proteobacteria* (11%) (Supplementary Fig. S3). The SWIB/MDM2 domain is also largely common in Eukaryota. This analysis suggests a common origin for the SWIB domain in the *Chlamydiae* phylum, whereas the possible eukaryotic origin of the SWIB domain in bacteria is still debated. The SWIB domain often co-occurs with the SET domain^41^, known to be also involved in chromatin remodelling^42^. Interestingly, a new chlamydial effector, NUE (Nuclear effector), is translocated into the host cell nucleus and possesses a SET domain. Importantly, NUE has been shown to methylate host cell histones but not bacterial histone-like proteins, i.e. chlamydial Hc1 and Hc2^42^.

### Wcw_0377 localizes in 293T nucleus

**Figure 7:**
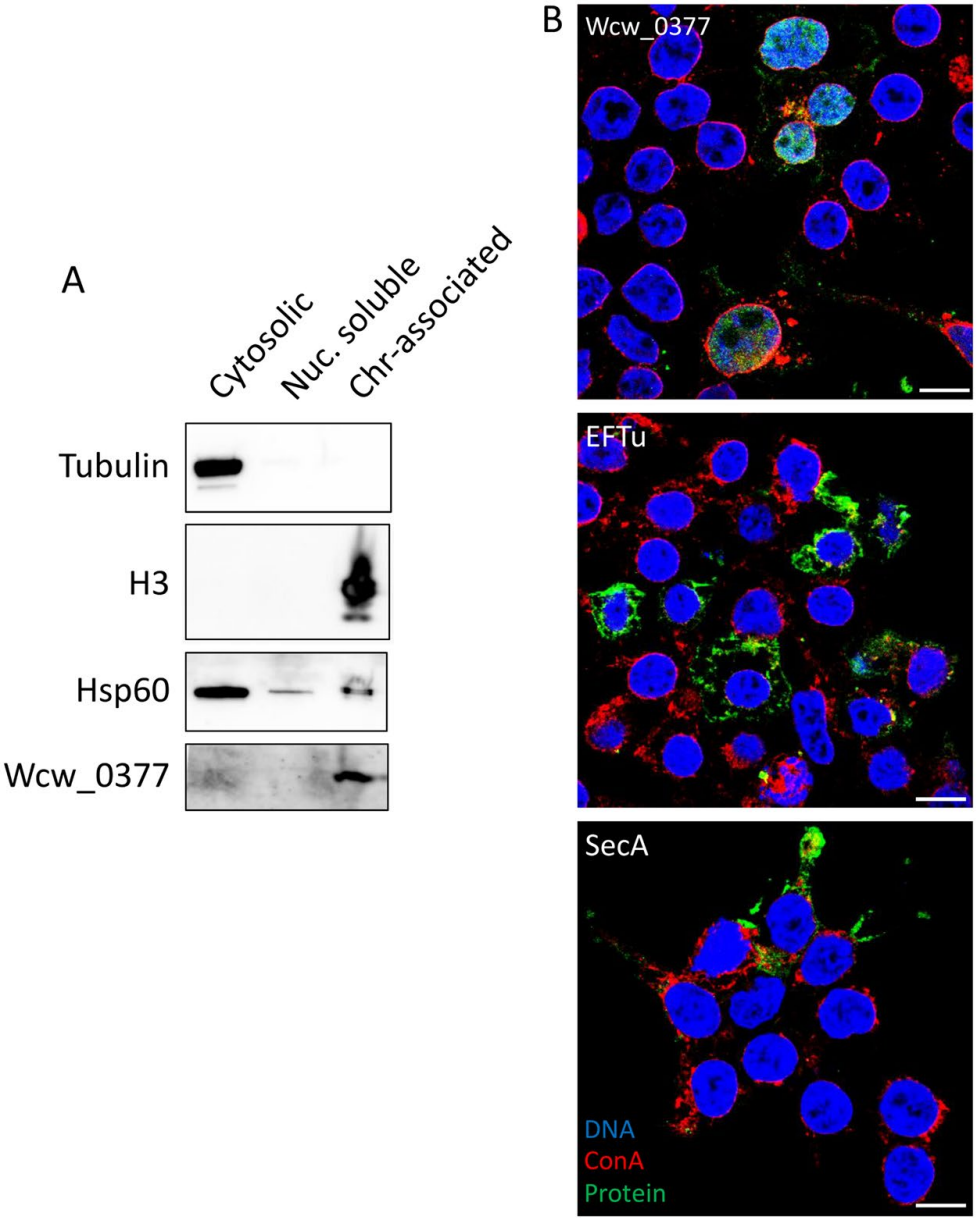
Wcw_0377 protein is translocated in host cell nucleus and is able to reach nucleusupon expression in eukaryotic cells. (**A**) Nuclear extraction was performed on infected Vero cells with *W. chondrophila*. Samples were analysed by immunoblotting using a polyclonal rabbit anti-tubulin, a polyclonal rabbit anti-histone H3, a polyclonal mouse anti-Hsp60 and a polyclonal mouse anti-Wcw_0377. Cytosolic fraction was diluted ten times before loading. As expected tubulin and histone H3 are only detected in the cytosolic and chromatin-associated fractions, respectively. Wcw_0377 is only detected in the chromatin-associated fraction, while Hsp60 is mainly present in the cytosolic fraction. (**B**) 293T cells were transfected with pcDNA-DEST47 carrying *wcw_0377*, *secA* or *eFTu* gene. Transfected cells were fixed 48 h after transfection and the different proteins detected by immunofluorescence using specific mouse polyclonal antibodies. DNA was stained with DAPI and glycoproteins using concanavalin A conjugated to TexasRed.

Despite the absence of shared conserved feature between NUE from *C. trachomatis* and Wcw_0377, the presence of the SWIB/MDM2 domain raised the possibility that Wcw_0377 could also localize in the host cell nucleus and co-localize with DNA. As shown by immunofluorescence, we did not observe any co-localization of Wcw_0377 with the Vero cell nucleus after infection with *W. chondrophila* (Fig. 3). However, this negative result could be due to a sensitivity problem. To clarify this point, we lysed *W. chondrophila* infected Vero cells 48 h p.i. and separated components of the cytosol from soluble nuclear proteins and chromatin. Wcw_0377 was highly enriched in the chromatin-associated fraction while *W. chondrophila* Hsp60 was mainly detected in the cytosolic fraction (Fig. 7A). As controls, we used tubulin and histone H3, which were mainly detected in the cytosolic and chromatin fraction, respectively, indicating that there was no cross-contamination between fractions. This result suggests that Wcw_0377 is secreted out of the inclusion and is able to reach the host cell nucleus. To determine whether translocation of Wcw_0377 to the host cell nucleus requires other bacterial factors, we expressed the *wcw_0377* gene from a transfection plasmid in non-infected 293T cells. SecA and EFTu were used as controls. We observed that in this system Wcw_0377 reaches the host cell nucleus and co-localize with the DNA, while SecA and EFTu exhibit a cytoplasmic localization (Fig. 7B). These observations suggest that despite the lack of classical nuclear localization sequence (NLS) Wcw_0377 is able to target and enter the host cell nucleus without excluding contribution of host cell factors.

## Discussion

In this study, we used an innovative approach combining heparin chromatography and mass spectrometry to identify new DNA-associated proteins of *W. chondrophila*. We further revealed the regulatory landscape of these proteins by ChIP-Seq. The main limitation of the heparin chromatography is that non-DNA-binding proteins can also bind to the negatively charged heparin. For this reason, we decided to focus on proteins harbouring a domain possibly involved in DNA binding. The aim of the ChIP-Seq was then to confirm DNA binding and identify the target regions. DNA binding could be either direct or indirect with other *Waddlia* proteins involved in the complex. Nevertheless, it will give new information on proteins associated to DNA that could be involved in the regulation of transcription or in the DNA structure and topology (which also play an important role for transcriptional regulation). Until recently, EBs were considered as inert particles, metabolically and transcriptionally inactive. However, recent studies on *C. trachomatis* showed that EBs are indeed able to respond to modifications in nutrient conditions. *W. chondrophila*, EBs biology, including transcriptional regulation and DNA structure and topology is not well understood. To fill this gap, we decided to focus this study on EBs rather than on RBs that are also less easily purified than EBs.

We studied three candidate proteins (Wcw_0377, Wcw_1456 and Wcw_1460) that co-localized with bacterial DNA. Their *in vivo* binding sites were identified by ChIP-Seq. Wcw_0377 emerged as a chromatin binding protein as suggested by its SWIB/MDM2 domain, whereas Wcw_1456 and Wcw_1460 harbour a wide regulon (762 and 902 regulated genes, respectively), including 691 co-regulated genes. Despite this large co-regulon, pull down experiments did not support any interaction between Wcw_1456 and Wcw_1460.

In addition, the size of the two regulons is in the range of sigma factors regulons. Accordingly, we demonstrated interaction between Wcw_1456 and RpoD (σ^66^) as well as between Wcw_1460 and RpoD, suggesting that these two proteins could act as regulators of RpoD. Unfortunately, in absence of mouse polyclonal anti-RpoD antibody and because of its insolubility, we were unable to confirm these interactions by pull-down or Co-IP experiments. Another way to confirm these interactions would be to compare the regulon of RpoD with those of Wcw_1456 and Wcw_1460 by ChIP-Seq. However, we cannot exclude that these interactions are indirect. Intriguingly, CT398, the homolog of Wcw_1456 in *C. trachomatis*, was shown to interact with RpoN as well as with two Type III secretion ATPase regulators, CdsL and FliH^36^. In *C. trachomatis*, the authors proposed that CT398 has a double function during infection, interacting with CdsL and FliH during the early phase of the developmental cycle and acting as post-translational RpoN chaperone during the late phase. Similarly, in *H. pylori* FlgZ harbours a fold (an N-terminal coiled-coil domain and a C-terminal C4-type Zn-ribbon) analogous to CT398 and Wcw_1456 and it was shown to interact with RpoN and FliH^43,44^. The absence of interaction between Wcw_1456 and Wcw_1460 with RpoN in our His pull-down experiments could be due to the fact that we used an EBs lysate. Thus, it could be interesting to perform His pull-down on purified RBs, despite the technical difficulties.

Intriguingly, the *C. trachomatis* homolog of Wcw_1460 (CT429) was described as secreted in the *Yersinia enterocolitica* heterologous system^37^. CT429 has no predicted function. Here, we showed that Wcw_1460 binds RpoD and regulates a large number of genes (directly or not). Nevertheless, we did not observe any secretion in the *Yersinia enterocolitica* heterologous system. Based on our results, we hypothesize that Wcw_1456 and Wcw_1460 form a complex with RpoD and are involved in its regulation, although the mechanism of RpoD regulation and the precise role of Wcw_1456 and Wcw_1460 in transcription initiation remain to be elucidated.

We demonstrated in this study that Wcw_0377 binds (directly or indirectly) all along the genome (1920 peaks), which is in accordance with the presence in this protein of a SWIB/MDM2 domain, known to be involved in chromatin remodelling. Wcw_0377 was detected in the host cell nucleus during infection and was able to target the nucleus when expressed in eukaryotic cells following transfection. However, in absence of a classical NLS (Nuclear Localization Sequence), the mechanism used by Wcw_0377 to reach the host cell nucleus remains undetermined. The exact function of Wcw_0377 both in the bacteria and in the host cell nucleus remains largely unexplored. As it features a putative chromatin-remodelling domain, it could be interesting to determine if Wcw_0377 possesses a methyltranferase activity towards the histone-like protein of *Waddlia* (Hc1) and/or eukaryotic histones, similarly to the chlamydial effector NUE^42^. If so, the identification of the host target genes as well as their expression profiles could be helpful to elucidate the impact of Wcw_0377 on the infection.

In *Chlamydiae*, the SWIB/MDM2 domain is found in two different proteins, a small one containing only the SWIB/MDM2 domain (Wcw_0377 and CT460) and a larger one containing a DNA topoisomerase I domain fused to the SWIB/MDM2 domain (Wcw_1713 and CT643). In addition, the SWIB/MDM2 domain often co-occurs with the SET domain (Wcw_0084 and CT737 NUE protein)^41^. The SET-domain proteins in eukaryotes are the primary methylases of lysines in the tails of histones H3 and H4^45,46^. The presence of SET, SWIB and topoisomerase I domains on these proteins strongly suggests that *Chlamydiae* possess a chromatin remodelling and modifying complex. Indeed, the chlamydial SET-domain protein NUE was already reported as a methylase of eukaryotic histones. For the methylation of the chlamydial histone-like protein Hc1, discordant results were obtained^42,47^.

Finally, since the SWIB and MDM2 domains share a common fold and MDM2 was described as an anti-apoptotic protein because it binds p53 inside the nucleus, we cannot exclude that Wcw_0377 is translocated inside the host cell nucleus to inhibit apoptosis^48^.

## Methods

### Bacterial strain and cell culture

*Waddlia chondrophila* strain ATCC VR-1470 was co-cultivated with *Acanthamoeba castellanii* strain ATCC 30010 at 32 °C, in 75 cm^2^ flask containing 30 ml of peptone-yeast-extract-glucose broth. After 7 days of co-culture, bacteria were isolated by filtering the suspension on 5 µm filter to eliminate trophozoites and cysts. The filtrate was then diluted at the appropriate dilution in DMEM to infect Vero cells.

Vero cells (ATCC CCL-81) were cultivated at 37 °C in the presence of 5% CO_2,_ in Dulbecco’s modified minimal essential medium (DMEM; GE Healthcare, Pasching, Austria) supplemented with 10% fetal bovine serum (FBS, GE Healthcare).

### Infection procedure

1 × 10^5^ Vero cells were seeded per well in 24-well plates and 1.2 × 10^6^ cells in a 25 cm^2^ flask, the day before infection (16 h before infection). A 1/2000 dilution of *W. chondrophila* was used to infect Vero cells, corresponding to an MOI of 2–3, as estimated by a *Waddlia* specific real-time quantitative PCR^49^. This led to 50% of infected cells with 2–3 bacteria per cell, as determined by confocal microscopy. The infection process was performed accordingly to de Barsy *et al*.^21^.

For Western blot analysis, infected cells were scraped and transferred in Eppendorf tubes. One hundred µl were used to extract genomic DNA and to proceed to real-time quantitative PCR using specific primers targeting the 16S rRNA region of *W. chondrophila* (detailed in de Barsy *et al*.^21^). Infected Vero cells were centrifuged at 12,300 *g* for 5 minutes and washed with 1 mL of PBS. Then cells were centrifuged again and resuspended in 500 µl of SDS sample buffer (60 mM Tris pH 6.8, 1% SDS, 1% mercaptoethanol, 10% glycerol, 0.02% bromophenol blue).

For immunofluorescence, infected Vero cells were washed once with PBS and then fixed by cold methanol for 5 minute or PFA 2% for 10 minutes.

### Heparin chromatography followed by mass spectrometry

One ml of purified EBs was resuspended in 2 ml of lysis buffer (20 mM Tris, 10 mM MgCl2, 1 mM EDTA, 1 mM DTT, 0.1% Triton X100) containing protease inhibitors, sonicated 30 seconds three times then supplemented with ready-lyse (Epicentre) and sonicated again. The lysate was centrifuged at 13,000 *g* for 15 minutes and the supernatant was collected and applied onto the heparin chromatography (GE Healthcare) pre-equilibrated with lysis buffer. The flow through was collected and the column washed with five volumes of lysis buffer. Proteins were eluted using five column volumes of elution buffer (lysis buffer with 1 M NaCl). The eluate was concentrated using Amicon ultra 5 K (Millipore, Cork, Ireland) according to manufacturer instructions. The soluble fraction of EBs and the eluate were sent to the Protein Analysis Facility (PAF) of the University of Lausanne for shotgun mass spectrometry analysis. Results were analysed using the MASCOT software directly by the facility.

For individual confirmation, His-tagged proteins were produced from *E. coli* Bl21 (DE3) pLysS containing pET28 vectors. Overnight cultures were diluted 1/50 in 50 ml of LB medium, induced with 0.5 mM IPTG when OD_600 nm_ reached 0.5–0.8 (Supplementary Table S6) and cultivated for 3 h at 37 °C. Induced cultures were centrifuged at 4000 *g* for 10 minutes. The pellets were lysed using 2 ml of lysis buffer (see above). Heparin chromatography (HiTrap Heparin HP, GE Healthcare, Uppsala, Sweden) was performed as described previously. Samples were separated on SDS-PAGE and transferred on nitrocellulose membrane for the immunoblotting (see above for details), using a monoclonal mouse anti-His_6_ (H1029, Sigma, St Louis, MO) at a 1/3000 dilution.

### Chromatin Immuno-precipitation followed by deep sequencing (ChIP-Seq) and ChIP-qPCR

ChIP-Seq experiment were carried out as detailed in de Barsy *et al*.^21^. We conducted 6 ChIP-Seq experiments using the three mouse polyclonal antibodies anti-Wcw_0377 (SZM610), anti-Wcw_1456 (SZM575) and anti-Wcw_1460 (SZM607)) and their corresponding preimmune sera. The preimmune sera were used to discard non-specific peaks during ChIP-seq analysis. Ten 25 cm^2^ flasks of Vero cells were infected per ChIP-Seq and recovered at 48 h p.i. for the immuno-precipitation. This late time point was chosen to ensure enough bacterial yields and bacterial DNA. The immunoprecipitated DNA was sent to Fasteris SA (Geneva, Switzerland) for library preparation and sequencing.

Specific primers pairs were designed to target selected regions identified by ChIP-Seq (Supplementary Table S7). We chose three shared targets and two specific targets for each protein among those with the highest peak shape score to design specific primer pairs. These primer pairs are located 75 nucleotides upstream and downstream of the peak center leading to 150 bp amplicon. The chromatin immunoprecipitation was performed as detailed above except that we used 500 µg of chromatin for each immunoprecipitation. After the preclearing step, we saved 10% as input chromatin and the remaining was divided in two (incubation with antibody and without antibody as mock control). The qPCR was performed as follows: the mixtures included 300 nM of each reverse and forward primer (Supplementary Table S7), 10 μL of iTaq universal SYBR green mix (BioRad, Hercules, CA) and 4 μL of DNA. The qPCRs were performed on the Step One PCR system (Applied Biosystems, Zug, Switzerland) using the following conditions: 3 minutes at 95 °C, 45 cycles of 15 seconds at 95 °C and 1 minute at 60 °C. A melting curve was then performed (15 seconds at 95 °C, 1 minute at 55 °C, +0.3 °C increment, 15 seconds at 95 °C). The amplification efficiency of each pair of primers, used at 300 nM, was determined and considered as good if comprised between 90 and 110%. For the data analysis, we used the input percentage method as described in Lin *et al*.^50^ and applied the following equations: ΔCt [normalized ChIP] = (Ct [ChIP] - (Ct [Input] - Log2 (Input Dilution Factor); Input% = 100/2 ^ΔCt [normalized ChIP]^.

### ChIP-Seq analysis

Libraries were prepared by Fasteris using the Illumina ChIP-Seq TruSeq kit. Single-end run were performed on an Illumina HiSeq2500 (1 × 50 bp) using the High Output mode and yielded several million reads. Bioinformatics analysis were done directly by Fasteris SA using the CLCbio genomic workbench. Reads were mapped against *Waddlia chondrophila* WSU 86–1044 genome leading to BAM files. Peaks were detected using the “ChIP-Seq analysis” module on CLCbio genomic workbench (peak shape score, p ≤ 0.01^51^). Preimmune sera were used as input samples and were taken into account to reduce noise. A quality control report was also supplied where quality measures were inferred based on the cross-correlation profile (Supplementary Table S8). Functional COG categories were attributed to each predicted coding sequence of the genome of *Waddlia chondrophila* (accession: NC_014225) based on the best BLASTP hit in the COG database (https://academic.oup.com/nar/article/43/D1/D261/2439462) using BLAST+ version 2.2.28+ (https://bmcbioinformatics.biomedcentral.com/articles/10.1186/1471-2105-10-421) with an e-value cutoff of 1e^−5^, a minimum query coverage of 50% and a minimum identity of 20%. We determined the occurrence (%) of each COG categories in each datasets (shared peaks, Wcw_1456 specific peaks and Wcw_1460 specific peaks).

### His pull-down assay

For the His pull-down assay, we used the Pierce Pull-Down PolyHis Protein:Protein Interaction Kit (Thermo Scientific, Rockford, IL) following the manufacturer’s instructions with minor modifications. Five ml of induced cultures of *E. coli* Bl21 (DE3) pLysS overexpressing His tagged Wcw_1456, Wcw_1460 or the His tag alone were lysed as recommended except that we added RNase, DNase and protease inhibitors. The soluble fraction was used in the next step. The HisPur Cobalt resin equilibration and the His tagged-proteins immobilization were done as described in the manual. For the prey protein preparation, we used one ml of purified EBs that we lysed as explained above and used 1/40 fraction per pull-down experiment. The elution of the protein complexes were directly done by addition of 60 µl of SDS sample buffer. The three samples were sent to the Protein Analysis Facility (PAF) of the University of Lausanne for shotgun mass spectrometry analysis. Results were analysed using the MASCOT software directly by the facility.

### Transfection

16 h before transfection, 1 × 10^5^ 293T cells per 24-well were seeded on glass coverslips in order to obtain 70% of confluence the day of the transfection. Cells were transfected with pcDNA-DEST47_*wcw_0377*/*secA/EFTu* using lipofectamine 3000 reagent following the manual’s instructions. Cells were fixed 48 h after transfection using 2% of paraformaldehyde for 15 minutes. Immunofluorescence was performed as detailed in the supplementary methods using polyclonal mouse antibodies anti-Wcw_0377 (SZM610, 1/200 dilution), anti-SecA (SZM601, 1/500 dilution) and anti-EFTu (SZM944, 1/500 dilution). Concanavalin A-Texas Red conjugate (diluted 1/50) was used to detect glycoproteins (cytoplasmic membrane and Golgi apparatus).

### Nuclear extraction

Nuclear isolation and separation of chromatin associated proteins from soluble nuclear proteins was performed as explained in Pennini *et al*.^42^. The three fractions (cytosolic, nuclear soluble and chromatin associated) were analysed by SDS-PAGE followed by immublotting (see above for details) using a polyclonal rabbit anti-histone H3 (1/1000 dilution, H0164, Sigma, St Louis, MO), a polyclonal rabbit anti-alpha tubuline (1/500 dilution, ab18251, Abcam, Cambridge, UK), a polyclonal mouse anti-Hsp60 (1/500, home-made) and a polyclonal mouse anti-Wcw_0377 (1/100 dilution, SZM610). Note that the nuclear soluble and chromatin-associated fractions were in an equal volume while the cytosolic fraction was in a 10 times higher volume.

## Supplementary information

Supplementary information

Supplementary Table S1

Supplementary Table S2

Supplementary Table S3

Supplementary Table S4

Supplementary Table S5
