# Publisher Correction: Identification of new DNA-associated proteins from *Waddlia chondrophila*

**DOI:** 10.1038/s41598-019-53160-y

**Published:** 2019-11-05

**Authors:** Marie de Barsy, Lucas Herrgott, Virginie Martin, Trestan Pillonel, Patrick H. Viollier, Gilbert Greub

**Affiliations:** 1 Institute of Microbiology, University Hospital Center and University of Lausanne, Lausanne, Switzerland; 2 Department Microbiology and Molecular Medicine, Institute of Genetics & Genomics in Geneva (iGE3), University of Geneva, Geneva, Switzerland

Correction to: *Scientific Reports* 10.1038/s41598-019-40732-1, published online 20 March 2019

In the original version of this Article, the author Marie de Barsy was incorrectly indexed. This error has now been corrected.

