# Supplementary material for: Identification of new DNA-associated proteins from Waddlia chondrophila
Source: Sci Rep. 2019 Mar 20;9:4885. doi: 10.1038/s41598-019-40732-1 (PMC6426960; doi:10.1038/s41598-019-40732-1)
Supplement: Supplementary file 1 — Supplementary information [file 41598_2019_40732_MOESM1_ESM.pdf]

Supplementary information:

Identification of new DNA-associated proteins from *Waddlia chondrophila*

Marie de Barsy[1], Lucas Herrgott[1], Virginie Martin[1], Trestan Pillonel[1], Patrick H. Viollier[2], Gilbert Greub[1*]

Supplementary methods

Immunoblotting

Immunobloting was performed as described in [1] with a slight modification in the detection step. The proteins were detected by chemoluminescence using ECL Prime Western Blotting Detection Reagents (GE Healthcare, Buckinghamshire, UK) and ImageQuant LAS 4000 Mini imager (GE healthcare, Waukesha, WI). Signals were quantified using ImageJ and normalized according to the number of bacteria per well. For each proteins, the maximum value was then normalized to 100% and the minimal value to 0%. Experiments were done in duplicate. Polyclonal mouse antibodies for Wcw_0377 (SZM610), Wcw_1456 (SZM575) and Wcw_1460 (SZM608) were used for immunoblotting at a 1/500 dilution.

Immunofluorescence

Methanol fixed Vero cells were permeabilized and blocked in blocking solution (0.1% saponin (Sigma-Aldrich), 0.2% NaN_3 (Acros, Geel, Belgium) and 10% FBS (GE Healthcare)) for 15 minutes at 37°. Coverslips were then incubated 1 h with an home-made polyclonal rabbit anti-*W. chondrophila* antibody (1/1000 dilution) and a polyclonal mouse anti-Wcw_1456 (SZM573, 1/500 dilution) or anti-Wcw_1460 (SZM607, 1/500 dilution) or anti-OmcB (SZM009, 1/20 dilution) or anti-Hsp60 (1/100 dilution) diluted in the blocking solution. Coverslips were washed 3 times in PBS and incubated with the secondary antibodies (Alexa-Fluor 488-conjugated goat anti-mouse IgG and Alexa-Fluor 594-conjugated goat anti-rabbit) diluted 1/1000 in the blocking solution, containing also DAPI (Molecular Probe, Eugene, Oregon, USA) at a concentration of 1μg/ml. After two washes with PBS and one with deionized water, coverslips were mounted with mowiol (Calbiochem, CA). Cells were observed under a confocal microscope (Zeiss LSM780).

For the immunodetection of the Wcw_0377, infected Vero cells were fixed with PFA, then permeabilized with Triton X100 0.5% for 5 minutes, washed 3 times with PBS and treated

with Image-iT FX signal enhancer (Molecular Probes, Eugene, OR) for 30 minutes. Cells were washed once with PBS and blocked in blocking solution (0.3% triton X100, 0.2% NaN_3 (Acros, Geel, Belgium) and 10% FBS (GE Healthcare)) for 15 minutes at 37°. After a last wash in PBS, fixed cells were treated as described above using polyclonal mouse antibody anti-Wcw_0377 (SZM610, 1/200).

Plasmids constructions, protein purification and antibodies production

The 9 genes were cloned in NdeI/EcoRI into pET28 except for the wcw_0677 (NheI/EcoRI) and wcw_1456 and _1581 (NdeI/SalI), as explained in [1]. These plasmids were used to transform *E. coli* Bl21 (DE3) pLysS allowing the overexpression after addition of IPTG. Overnight cultures were diluted 1/50 in LB medium, when OD_{600nm} reached 0.5-0.8, cultured were induced by addition of 0.5 mM of IPTG and gowned for 3h at 37°C. His_6-Wcw_0377 and His_6-Wcw_1460 were purified on Ni-NTA agarose (Qiagen, Hombrechtikon, Switzerland) under native conditions following the manufacturer's instructions, while His6-Wcw_0150 and His6-Wcw_1456 were purified using MagneHis Ni-particle (Promega, Madison, WI) following manufacturer's instructions. 180 µg of each purified proteins were used to immunize three mice with the classical protocol for custom polyclonal antibodies of Eurogentec (Liège, Belgium).

The pcDNA_DEST47 is a transfection plasmid allowing constitutive high expression in mammalian cells under the CMV promoter. This plasmid also carry a C-terminal cycle 3 GFP and is compatible with Gateway cloning. To amplify our genes of interest, we used primer compatible with the Gateway technology, meaning that they harbour attB1 and attB2 recombination sites on the forward and the reverse primers, respectively (Supplementary Table S9). To allow expression of our genes in mammalian cells, we added the Kozak sequence at the forward primer. To only express our genes and not the fusion to the GFP, we keep the stop codon of our genes of interest. After amplification, the PCR products were

inserted into the pDONR201 by BP reaction, followed by LR reaction to insert our genes of interest into the pcDNA_DEST47.

Taxonomic distribution of the SWIB/MDM2 domain (PF02201) in representative genomes

The Pfam domain PF02201 was identified in 6661 reference and representative genomes available from RefSeq (September 2017) using hmmsearch (HMMER version 3.1b2, http://journals.plos.org/ploscompbiol/article?id=10.1371/journal.pcbi.1002195). Hits were filtered based on PFAM trusted cutoffs. Genomes were then grouped by phylum based on the NCBI Taxonomy database (https://academic.oup.com/nar/article/40/D1/D136/2903327) with the python library ete2 (https://bmcbioinformatics.biomedcentral.com/articles/10.1186/1471-2105-11-24). Only phyla exhibiting a minimum of 5 genomes were reported in Supplementary Fig. S3.

Supplementary figures

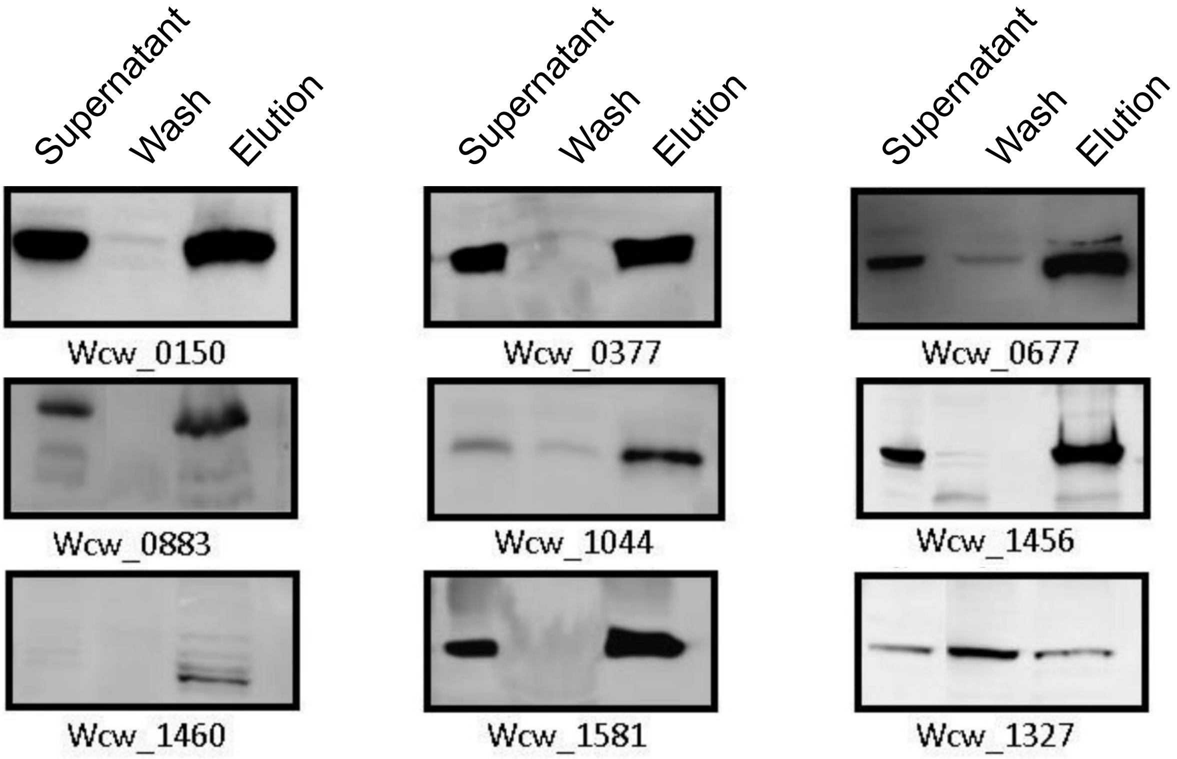

Supplementary figure 1. Individual confirmation by heparin chromatography. Heparin chromatography was performed on the eight His-tagged protein produced in *E. coli*. The soluble fraction, the wash and the elution were collected, concentrated and analysed by SDS-PAGE followed by immunoblot using monoclonal mouse anti-His_6. As a control, we used the His_6-Wcw_1327. All the eight proteins were enriched in the elution in comparison to the control Wcw_1327, which is enriched in the wash fraction.

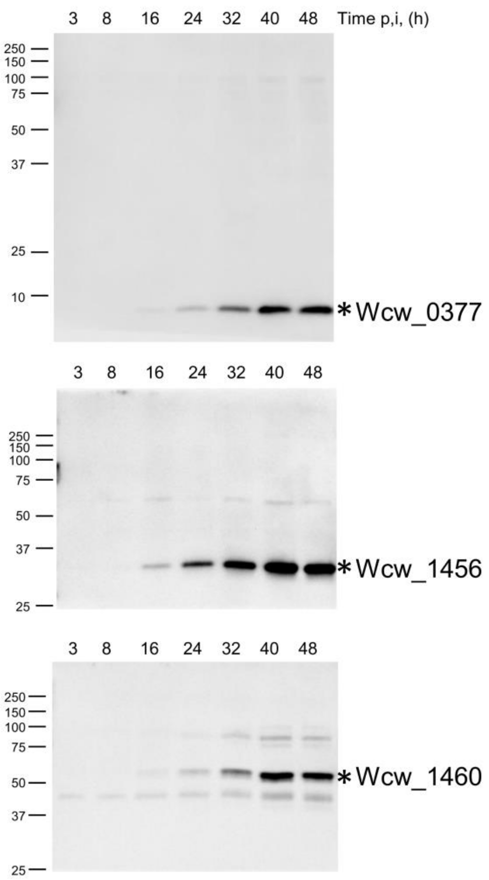

Supplementary figure S2. Full-size blot pictures for the temporal expression the three *Waddlia* proteins during the developmental cycle. Samples of infected Vero cells were collected at different times p.i. and analysed by immunoblotting using specific mouse polyclonal antibodies.

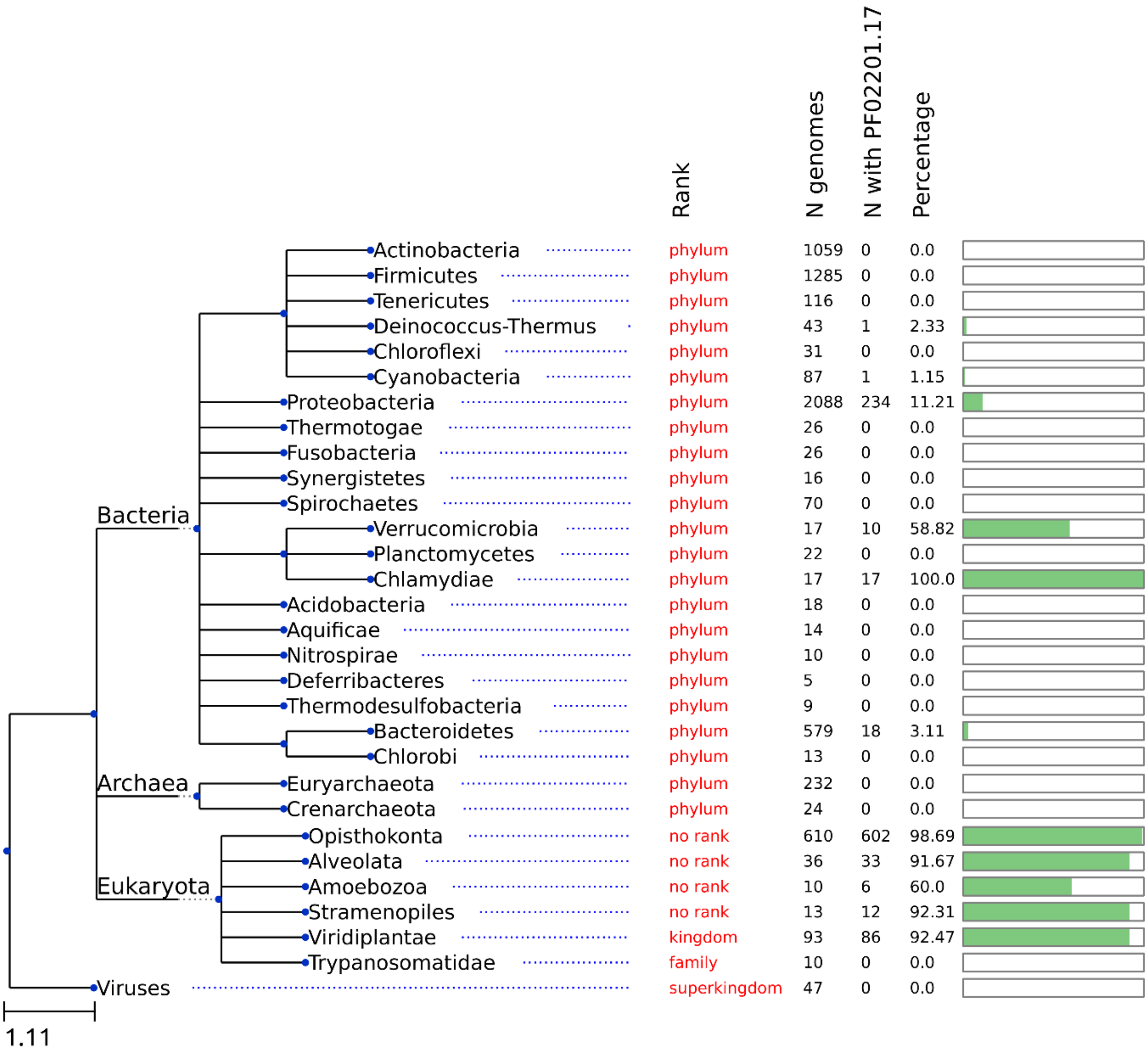

Supplementary figure S3. Presence of the SWIB/MDM2 domain (PF02201) in 6626 genomes. This domain is present in 100% of bacteria belonging in the *Chlamydiae* phylum while it is detected in 60% of Verrucomicrobia and in 11% of Proteobacteria. It is also commonly present in Eukaryota.

Supplementary tables

Supplementary Table S1. Mass spectrometry after heparin chromatography on the *W. chondrophila* EBs soluble fraction. In red character, the 132 enriched proteins with a fold change ≤ 0.5 with 95% confidence, highlighted in green the 9 selected proteins.

Supplementary Table S2. Selection of 9 hypothetical proteins potentially associated to DNA.

Supplementary Table S3. ChIP-Seq data for the Wcw_0377, _1456 and _1460. For each data set, we have the region, the peak center, the length of the peak, the peak shape score and the P-value.

Supplementary Table S4. Annotated peaks for Wcw_1456 and _1460. Each peak was assigned to its potentially regulated gene(s) (-400 nt < max peak < + 100 nt, 0 position = gene start). Sheat 1. Shared peaks between Wcw_1456 and _1460. Sheat 2. Specific peaks of Wcw_1456. Sheat 3. Specific peaks of Wcw_1460. **.** Highlighted in green assigned peaks used for ChIP-qPCR.

Supplementary Table S5. His pull-down coupled to mass spectrometry. Highlighted in green enriched protein compared to negative control, in orange possible cross-contamination.

Supplementary Table S6. IPTG induction conditions to produce His-tagged proteins in *E. coli* Bl21 pLysS ou C41 strains.

Proteins	*E. coli* strains	Plasmids	Molecular weight	Conditions of induction
Wcw_0150	Bl21 (DE3) pLysS	pET28a	29 kDa	0,5 mM IPTG, 37°C 3h
Wcw_0377	Bl21 (DE3) pLysS	pET28a	10 kDa	0,5 mM IPTG, 37°C 3h
Wcw_0677	C41	pET28a	87 kDa	0,5 mM IPTG 37°C 3h
Wcw_0883	Bl21 (DE3) pLysS	pET28a	12 kDa	0,5 mM IPTG, 37°C 3h
Wcw_1044	Bl21 (DE3) pLysS	pET28a	25 kDa	0,5 mM IPTG, 37°C O/N
Wcw_1456	Bl21 (DE3) pLysS	pET28a	30 kDa	0,5 mM IPTG, 37°C 3h
Wcw_1460	Bl21 (DE3) pLysS	pET28a	47 kDa	0,5 mM IPTG, 37°C 3h
Wcw_1581	Bl21 (DE3) pLysS	pET28a	106 kDa	0,5 mM IPTG, 25°C O/N

Supplementary Table S7. Primers used for the ChIP-qPCR.

Primer name	Sequence	Regulon
Pwcw_RS00005_fwd	GACTTGTGAGATATGATCAAAAGTTGTGTTTGAG	Wcw_1460
Pwcw_RS00005_rev	GCTGTTCATTTCACTTGTTGCGTGAAATTTATC	
Pwcw_RS02540_fwd	GGTCAAGATCAGCAGCTACGAATTGAG	Shared
Pwcw_RS02540_rev	ACTGGGATGCGAAGTATTGATTGCATGC	
Pwcw_RS02805_fwd	GAACTGAAAGTGTATACAAAAGTTGTTGTCGCTT	Wcw_1456
Pwcw_RS02805_rev	GTTTATCCTTATCTTTCTCTAGCCGGTAATTAAC	
Pwcw_RS04290_fwd	CATAGCTCAACTGGTAGAGCACCCGA	Shared
Pwcw_RS04290_rev	TCGAACCAACGTAGACCGAAGTCGAG	
Pwcw_RS08450_fwd	ATTGAACTGATACAAGAATATTTAATTAAGCCACAAGAC	Wcw_1460
Pwcw_RS08450_rev	TCCGATTCTTGAAGTTGATTCGGTATAAATTTAATTATTTTT	
Pwcw_RS07245_fwd	TTTATCGGCTTTTAACCGCACACTTGTTATTG	Shared

Pwcw_RS07245_rev	TTTTAGAAATGAAAGCTGAATGCAAAGGTCCCTT	
Pwcw_RS05030_fwd	ATCGTTGAAAAAACGATGATGCAAATCACAGATC	Wcw_1456
Pwcw_RS5030_rev	CAACAGAACGACCTCTCGATCTCTGT	
PhrcA_CTL_fwd	GCTGCTCAATTGGTCTATAAAAAAGGACTTGA	HrcA
PhrcA_CTL_rev	TTTCATTATAACAAGGGGTTTAAAAGAGGTCAAGAAG	
Pwcw_RS02005_fwd	GGATGAATGGAGACACTAATATTGTTTCTATGG	/
Pwcw_RS02005_rev	GATTTCAGTCACGAGATTTCCGCATCCAA	

Supplementary Table S8. ChIP-Seq quality control report.

Sample	Number of reads	Relative strand correlation (>0.8)	Normalized strand coefficient (>1.05)
anti_1456	4938353	0,988	1,086
Preimmune anti_1456	1818405	0,238	1,082
anti_1460	8436580	1,383	1,063
Preimmune anti_1460	1465716	0,088	1,037
anti-0377	9770942	0,455	1,017
Premimmune anti_0377	1737872	0,165	1,073

Supplementary Table S9. Primers used the pcDNA-DEST47 constructions.

Name	sequence (5'-3')
EFTu_attB1_Kozak	GGGGACAAGTTTGTACAAAAAAGCAGGCTCGACGATGGCGAAAGAAACATTTCAAAGAAATAAGC
EFTu_attB2_stop	GGGGACCACTTTGTACAAGAAAGCTGGGTATTACTCAATAATTTCGGAAACTGTTCCTGCT
wcw_0377_attB1_Kozak	GGGGACAAGTTTGTACAAAAAAGCAGGCTCGACGATGGCAAAACAAAAAAAACCATCAGCGTTC
wcw_0377_attB2_stop	GGGGACCACTTTGTACAAGAAAGCTGGGTATTATTTAGCACCCGCCGCTTCAG
secA_attB1_Kozak	GGGGACAAGTTTGTACAAAAAAGCAGGCTCGACGATGGTCAGTTTTTTCAAGAAATTATTCGGAACAG
secA_attB2_stop	GGGGACCACTTTGTACAAGAAAGCTGGGTATTATTCGTCAGCCTGATGGATGCC

References

1. de Barsy, M. *et al.* Regulatory (pan-)genome of an obligate intracellular pathogen in the PVC superphylum. *ISME J* **10**, 2129-44 (2016).
